# West Nile virus: The current situation in Egypt

**DOI:** 10.14202/vetworld.2023.1154-1160

**Published:** 2023-05-30

**Authors:** Rabab T. Hassanien, Heba A. Hussein, Hala K. Abdelmegeed, Dina A. Abdelwahed, Omnia M. Khattab, M. H. Ali, Ahmed R. Habashi, Essam M. Ibraheem, Momtaz A. Shahein, Eman M. Abohatab

**Affiliations:** 1 Department of Virology, Animal Health Research Institute, Agriculture Research Center, 12618, Giza, Egypt; 2 Genome Unit, Animal Health Research Institute, Agriculture Research Center, 12618, Giza, Egypt; 3 Virus Strain Bank, Animal Health Research Institute, Agriculture Research Center, 12618, Giza, Egypt; 4 Department of Pathology, Animal Health Research Institute, Agriculture Research Center, 12618, Giza, Egypt

**Keywords:** climatic changes, flaviviruses, seroprevalence, West Nile virus

## Abstract

**Background and Aim:**

Due to climatic changes, arthropod-borne viruses have become a global health concern. In Egypt, West Nile virus (WNV) was initially detected in humans in 1950 and then in 1951, 1954, 1968, and 1989. Although WNV infection has been recorded in numerous Middle Eastern countries, its prevalence among the equine population in Egypt is unknown. This study aimed to investigate the current situation of vector-borne WNV in Egypt, estimate its seroprevalence, and assess the associated risk factors.

**Materials and Methods:**

We screened 1100 sera samples and nasal swabs from the same equids, 156 mosquito pools, and 336 oropharyngeal and cloacal swabs from migratory birds for WNV. The sera were investigated for the presence of immunoglobulin G (IgG) and immunoglobulin M (IgM) against WNV-prE. Real-time reverse transcription-polymerase chain reaction was used to detect WNV RNA in the nasal swab samples, mosquito pools, and migratory birds’ oropharyngeal and cloacal swabs.

**Results:**

The seroprevalence showed positive IgG in sera samples collected from different districts. The data showed that horses were 1.65-fold more susceptible than donkeys, with male being 1.45 times more susceptible than females. Moreover, the tested equids samples were divided into three groups based on their age: <5 years, 5–10 years, and >10 years. The 5–10-year group was 1.1 and 1.61 times more vulnerable to infection than the <5- and >10 year groups. All the sera samples were negative for IgM. The nasal swabs from equids, oropharyngeal and cloacal swabs from migratory birds, and mosquito samples tested negative for WNV by molecular detection.

**Conclusion:**

Based on the obtained data, we recommend that effective control programs should be implemented to enable epidemiological investigations and understand the current situation of WNV in Egypt.

## Introduction

Flaviviruses are spread extensively worldwide and include the West Nile virus (WNV), Japanese encephalitis virus, Zika virus (ZIKV), Yellow fever virus, mosquito-borne dengue virus, tick-borne encephalitis virus, and Usutu virus [1, 2]. West Nile virus is an emerging zoonotic virus that can infect humans, horses, and several bird species. According to the World Organization for Animal Health, West Nile fever (WNF) seriously impacts livestock and public health [3]. It is an arthropod-borne virus naturally maintained in *Culex*, including *Culex* *univittatus* and *Culex* *pipiens*, which are ornithophilic mosquitoes that feed on birds. The mosquitoes are virus vectors, whereas the birds are virus reservoirs. The virus replicates in birds and mosquitoes and is transferred to dead-end hosts, such as horses and humans [4]. The infection is often asymptomatic or moderate in humans, but in infected horses, symptoms can vary from mild ataxia and muscular weakness to severe ataxia and recumbency [5].

This virus is an RNA-based *Flavivirus* belonging to the family *Flaviviridae*. It is a spherical-shaped and enveloped virus with an 11-kb positive-sense single-stranded RNA genome. The translated viral polyproteins consist of three structural (C, prM/M, and E) and seven non-structural (NS1, NS2A, NS2B, NS3, NS4A, NS4B, and NS5) proteins [6]. Genetic and phylogenetic analyses revealed that WNV has two lineages: 1 and 2. Lineage 1 includes three sublineages: a, b, and c. Lineage 1a circulates in Africa, America, Europe, and the Middle East, Lineage 1b (known as Kunjin virus) is found in Australia, and Lineage 1c is mainly distributed in India. The Sub-Saharan African strains belong to WNV Lineage 2. Other possible WNV lineages have been previously reported, such as Lineage 3 (Rabensberg virus) in the Czech Republic and Southern Moravia, Lineage 4 in Russia, Lineage 5 in India, putative Lineage 6 in Spain, Lineage 7 (Koutango virus) in Koutango, Senegal, and later in Somalia, putative lineage 8 virus was isolated from *Culex perfuscus* in Kedougou, Senegal, and Lineage 9 in Austria [7–9]. Serological diagnosis is often used to detect WNV infection. West Nile virus antibodies can be detected in domestic animals using various serological procedures, including enzyme-linked immunosorbent assays (ELISA), immunofluorescence assays, and viral neutralization tests [10, 11].

Recently, the epidemic spillover and spread of the flaviviruses have been geographically distinguished [12]. For instance, significant WNV and ZIKV infections have been reported in humans, especially in the Western population [13, 14]. Moreover, WNV outbreaks have been reported in France, Italy, Greece, South Africa, Hungary, Southeast Romania, and the USA [15]. Furthermore, WNV was found in several Middle Eastern and Asian countries, including Jordan, Palestine, Israel, Iran, Saudi Arabia, and Turkey [16, 17]. In Egypt, WNV infection was reported for the first time in 1950 in Northern Cairo. Later, several outbreaks occurred between 1952 and 1954 [18]. A cohort study reported human WNV seroprevalence in Egypt using WNV isolated from sentinel chickens and mosquitoes, indicating the active circulation of WNV in Egypt [19]. More recently, a serological survey for WNV detected this virus in equids in the Northern Egyptian Governorates, mainly in Qalyubia and Kafr El Shiek [20].

As WNV are arthropod-borne viruses, they are affected by climatic changes that also impact the future emergence of zoonotic viral diseases. Climate change has caused global temperature fluctuations and unpredictable precipitation patterns, contributing to the spread of mosquito-borne arboviruses and the mosquito populations that transmit them [21]. These viruses can potentially assume new hosts, increasing the risk of zoonotic diseases in humans [14]. The epidemiological features of arthropod-borne viruses may be affected by climatic changes, insect vectors, and their geographical distribution [13]. Therefore, assessing the status of WNV, a major arthropod-borne virus in Egypt is crucial.

This study aimed to investigate the current situation of WNV in Egypt, estimate its seroprevalence and evaluate the risk factors from 2020 to 2022. This information is critical for successfully implementing viral prevention and control programs in Egypt.

## Materials and Methods

### Ethical approval

The Local Ethics Committee of Animal Experiments at the Animal Health Research Institute (AHRI), Agriculture Research Center (ARC), Egypt (ARC-AHRI-23-02), has approved the sample collection in this study following institutional, national, and international guidelines.

### Study period and location

A total of 1100 samples were collected in 2020, 2021, and 2022 (400, 600, and 100 samples, respectively), from imported and native animals from different governorates (i.e., Cairo, Giza, El-Mnofiya, Al-Qalyoubia, El-Menia, El-Zagazig, Alexandria, Port-said, El-Fayoum, Aswan, Ismailia, Hurghada, and Al Arish) (supplementary data).

### Sample collection and preparation

#### Sera samples

A total of 1100 sera samples were collected from equid species (500 samples from horses and 600 from donkeys). These included samples from 732 male and 368 female specimens aged <5 years (n = 203), 5–10 years (n = 545), and >10 years (n = 352). The blood samples were collected from the jugular vein in clean, dry centrifuge tubes, allowed to clot, and centrifuged at 1500× *g* for 20 min to obtain the serum. The serum samples were kept at −20°C and used to detect antibodies against WNV (immunoglobulin [Ig]G and IgM).

#### Swab samples

For nasal swabs, 1100 samples were collected from the same animals, ages, and governorates as described above (supplementary data). Each swab was transported in 0.75 mL of sterile phosphate buffer saline (PBS; 0.01 M pH 7.4), vortexed vigorously for 10 min, and centrifuged for 5 min at 2000× *g*. Viral RNA was extracted from this supernatant for detecting WNV nucleic acid.

#### Mosquito samples

We collected 156 mosquito pools to detect WNV (i.e., 52 pools/each year; 4 groups/governorate) (supplementary data). CDC miniature-light traps (developed by Centers for Disease Control, USA) baited with CO_2_ were used to trap mosquitoes. The traps were placed in rural and urban locations adjacent to water sources and/or near animals. After transporting the collected mosquitoes to the laboratory in closed and chilled containers, they were counted and pooled according to the date and location. The mosquitoes were then ground with sterile sand using RNA lysis buffer in a sterile mortar. The lysates were centrifuged at 24,500× *g* for 10 min at 4°C to clarify the homogenates. The supernatant from the mosquito homogenate was transferred to a 1.5 mL microfuge tube and used for RNA extraction for detecting WNV nucleic acid [20].

#### Migratory birds

Oropharyngeal and cloacal swabs (i.e., 336 samples) were collected from egrets in the years 2020 (n = 126), 2021 (n = 156), and 2022 (n = 54) from different Egyptian Governorates (Supplementary data). The samples were prepared as mentioned above and used to detect WNV nucleic acid.

### Detection of antibodies against WNV

The prepared sera were tested using ID Screen® West Nile Competition Multi-species ELISA Kit (IDvet, rue Louis Pasteur, Grabels, France) to detect anti-prE IgG in the sera samples according to the manufacturer’s instructions [22].

Furthermore, the same sera samples were tested to detect anti-prE antibodies IgM using ID Screen® West Nile IgM Capture ELISA kit, IDvet. Immunoglobulin M (IgM) antibodies indicate recent infection and WNV circulation [3, 23].

### Molecular detection of WNV

The viral RNA was extracted using QIAamp Viral RNA Mini Kit (250) (Qiagen, Valencia, CA, Cat. no. 52906). Then, the extracted genome was subjected to real-time reverse transcription-polymerase chain reaction (RT-PCR) (genesig^®^ Standard RT-PCR detection kit for WNV, REF#(Z-PathWNV-std)). Based on the manufacturer instructions, 20 μL reaction volume contained 10 μL One Step or Precision PLUS One Step 2× RT-qPCR Master Mix, 1 μL WNV primers and probe mix, 4 μL RNAse-free water, and 5 μL RNA template. Following the thermal profile, RT-PCR was performed in the Step OnePlus™ RT-PCR system (Applied Biosystems, Waltham, Massachusetts, USA, Cat. No.: 4376600). Reverse transcription was performed at 55°C for 10 min, enzyme activation was performed at 95°C for 2 min, and then, 40 cycles of denaturation, annealing, and extension were performed at 95°C for 10 min and 60°C for 60 s.

### Statistical analysis

All data are presented as mean ± standard deviation. The results were analyzed, and statistical significance was determined using the Chi-square and statistical hypothesis tests. p < 0.05 was considered statistically significant (Supplementary data).

## Results

### Serosurveillance for screening the virus

Serosurveillance for WNV was performed using 1100 sera samples obtained from all four quarters of Egypt. The results showed that 293 samples (26.6%) were positive and 807 samples (73.4%) were negative for the WNV-IgG antibodies (Table-1). The yearly distribution for all 293 positive samples was as follows: 101 samples in 2020 (34.5%), 180 samples in 2021 (61.4%), and 12 samples in 2022 (4.1%). These positive samples included 160 (54.6%) and 133 (45.4%) samples from horses and donkeys, respectively. Regarding the sex of the tested animals, we tested 732 males and 368 females, of which 212 (72.4%) and 81 (27.6%) positive samples and 520 (64.4%) and 287 (35.6%) negative samples belonged to males and females, respectively.

**Table-1:** Results of WNV-IgG antibodies in the collected sera samples during the 3 years’ study period.

Data of collected samples	No. of collected samples	WNV IgG		% of positive/total positive	odds ratio	CI	P-value
	
2020, 2021, 2022		Positive	Negative				
Governorate							
Imported	52	17	35	5.8%			
Giza	98	31	67	10.6%			
Cairo	81	30	51	10.2%			
El-Mnofiya	73	23	50	7.8%			
El-Kalyoubia	72	18	54	6.1%			
El-Menia	83	24	59	8.2%			
El-Zagazig	90	24	66	8.2%			
Alexandria	67	13	54	4.4%			
Port-said	81	17	64	5.8%			
El-Fayoum	78	17	61	5.8%			
Aswan	95	20	75	6.8%			
Ismailia	84	17	67	5.8%			
Hurghada	64	16	48	5.4%			
Al Arish	82	26	56	8.9%			
Total	1100	293	807	-			
Sex							
Male	732	212	520	72.4%			
Female	368	81	287	27.6%	1.45	1.08-1.94	<0.025
Breed							
Horse	500	160	340	54.6%			
Donkey	600	133	467	45.4%	1.65	1.26-2.16	<0.001
Age							
< 5 years	203	58	145	19.8%			
5-10 years	545	165	380	56.3%	0.92	0.65-1.31	<0.75
> 10 years	352	70	282	23.9%	1.61	1.08-2.41	<0.025

CI=Confidence interval, WNV IgG=West Nile virus immunoglobulin G

Regarding age, the results of the antibody screening against WNV showed that 58 (19.8%), 165 (56.3%), and 70 (23.9%) samples from animals in the <5 year-, 5–10 year-, and >10 year groups, respectively, tested positive for WNV (Table-1). Moreover, all sera samples obtained from equids tested negative for IgM against the WNV-prE antigen.

### Epidemiological map

**Figure-1:**
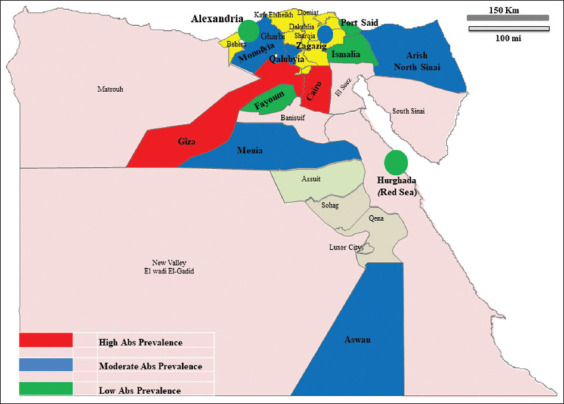
Map showed the geographical distribution of the serosurveillance results carried out in different Egyptian governorates. The red color indicated a high antibody prevalence (>10%), the blue color indicated a moderate prevalence (6–10%), and the green color indicated a low antibody level (<6%) based on the investigated immunoglobulin G levels in the tested sera samples [Source: https://www.maps.google.com].

The samples of this study were collected from different governorates all over the country. Thus, the serosurveillance of these districts represents the epidemiological situation of the disease in Egypt. Overall, the situation was involved after testing the collected sera samples for the presence of WNV-IgG and IgM antibodies and investigating the migratory birds (i.e., egrets) and the seasonal mosquitoes (i.e., Culex). The IgM antibodies weren’t detected in the tested sera samples. In addition, the migratory birds (i.e., egrets) and the seasonal mosquitoes (i.e., Culex) were negative for WNV nucleic acid as per the molecular screening. Hence, based on the obtained IgG results, the prevalence of the disease in different governorates is shown in the map in Figure-1.

### West Nile virus risk assessment analysis

The risk analysis for West Nile fever disease in Egypt was performed based on the obtained data from the serological investigation for IgG. This analysis determined the correlation between the species, sex, and age in the presence of WNV-IgG. The risk of the disease prevalence was 1.65 times more in horses than in donkeys. Furthermore, male horses were 1.45 times more susceptible than females. Based on the tested animals’ age, the samples obtained from animals aged 5 to 10 years were 1.1 and 1.61 times more susceptible to the virus than those aged <5 years and >10 years, respectively.

### Molecular screening for WNV

Molecular investigation was performed on the 1100 swab samples obtained from horses and donkeys, 156 mosquito pools, and 336 oral and cloacal swab samples from migratory birds to detect WNV. All the samples tested negative.

## Discussion

Mosquito-borne viruses significantly affect public health. West Nile virus, a *Flavivirus*, is a potential global health concern. The virus was initially identified in Egypt in 1951, and subsequent epidemics were documented in 1954, 1968, and 1989. Several studies have shown the circulation of WNV in Egypt [19, 20].

Furthermore, vector-borne diseases are significantly affected by the vector’s habitat and climatic and environmental changes, which directly influence the epidemiology and distribution of these diseases. Due to the severity and rapid spread of flaviviruses, they have significant global concern. Therefore, herein, we investigated the seroprevalence of WNV in horses and donkeys (dead-end hosts), insects, and birds (as vector hosts) to understand its current situation and predict the disease incidence and risk.

Fourteen different districts were included in this study conducted from 2020 to 2022. Significantly, the risk was estimated based on the obtained data. We detected IgG antibodies against WNV in 293 samples out of 1100 sera samples (26.6%) using ELISA, which can be attributed to previous infections or cross-reactivity between related flaviviruses in these animals [24]. West Nile virus-IgG antibodies might persist inside susceptible hosts for a long time due to early immune system activation that produces long-term antibody memory, which explains the presence of WNV-IgG in this study [25].

A previous study showed that the seroprevalence of WNV was 16.8% in Egypt, 15.08% in Poland, 15% in Portugal, 39% in Israel, 24.9% in Jordan, 26.8% in Algeria, 31.1% in Morocco, 31.6% in Turkey, and 68.7% in Senegal [26]. In this study, no significant difference in the seroprevalence of WNV in the investigated localities was detected, consistent with the previous study of Selim and Abdelhady [4]. However, Giza (10.6%) and Cairo (10.2%) showed the highest seroprevalence of WNV. As the climate is a potential factor for disease distribution, this can be attributed to the high temperature and humidity during summer in these areas.

Immunoglobulin G antibodies were detected in 54.6% and 45.4% of the sera samples from horses and donkeys, respectively, indicating a higher seropositivity rate in horses than in donkeys. The susceptibility was 1.65 times higher in horses than in donkeys (odds ratio [OR] 1.65, confidence interval [CI] 1.26–2.16; p < 0.001), probably because donkeys are more tolerant to harsh climatic changes and more disease-resistant than horses [4].

The age of the animals under investigation was considered in the study to estimate the disease risk rate among different age groups of the animals. For instance, three groups of age ranges were included, <5 years, 5–10 years, and >10 years. The highest seroprevalence (56.3%) was observed in the middle age group (i.e., 5–10 years). Statistical analysis revealed that 5–10 year old animals were 1.1 times more sensitive to disease than <5 year old animals (OR = 0.92, CI = 0.65–1.31, p < 0.75). Moreover, the animals in the <5 year group were 1.61 times more susceptible than those in the >10 year group (OR = 1.61, CI = 1.08–2.41, p < 0.025). This finding could be assigned to cumulative viral exposure over time [26]. However, the previous studies have not shown any relationship between the age of the animal and the seropositivity for WNV [27, 28].

In addition, the recorded seroprevalence for WNV exposure was higher among males (72.4%) than females (27.6%), indicating that males are 1.45 times more susceptible than females (OR = 1.45, CI = 1.08–1.94, p < 0.025). This finding came in agreement with a prior study performed by Epp *et al*. [29]. Although we could not identify a clear reason for this, it might be due to the higher stress on males who work harder than females. Stallions have poorer immune responses than mares or geldings because testosterone impairs immune functions [26].

Throughout the study, the highest seroprevalence was detected in 2021 (61.4%), followed by 2020 (34.5%) and 2022 (4.1%). This might be due to the larger sample size collected in 2021 (600 samples) than in 2020 (400 samples) and 2022 (100 samples). Overall, there was no significant difference in seroprevalence among the 3 years.

Immunoglobulin M antibodies indicate potential viral infection as it results from a humoral response to recent infections and is detectable 5–7 days post-infection [30, 31]. The ELISA results revealed that all sera samples were negative for WNV (IgM) antibodies. Based on this, we can conclude that there were no current active WNV infections in the animals tested from different localities in Egypt.

Migratory birds are major WNV vectors in countries South of the Sahara Desert to North Africa and Europe [32]. As mosquito-borne viruses are a significant public health concern worldwide [33], detecting these viruses in mosquito species should be further studied to clarify the role of WNV [34]. Hence, we used real-time PCR to detect WNV in different samples (nasal swabs from horses and donkeys, oropharyngeal and cloacal swab samples of migratory birds, and mosquito samples). All samples tested negative for WNV.

## Conclusion

The negative real-time PCR results agreed with the serological detection of IgM against WNV. Based on this, we concluded that no WNV strains are currently circulating in Egypt. Although the detected IgG antibodies might result from past infections or cross-reactivity between related flaviviruses, these results potentially indicate the current disease status and incidence, which requires further research. It is suggested to establish an immediate implementation of intensive surveillance and disease control by the seasonal sample collections from birds, mosquitoes, and horses to stand on the epidemiological situations of the virus in the country.

## Data Availability

The supplementary data can be available from the corresponding author on a reasonable request.

## Authors’ Contributions

RTH: Performed the experiments, data analysis, writing, editing and revision of the manuscript. HAH: Conceptualization, performed the experiments, data analysis, wrote, edited and revised the manuscript. HKA and DAA: Performed the experiments and wrote the original draft. OMK: Prepared the samples and performed the molecular investigation. MHA: Collected the samples and revised and edited the original draft. ARH, EMI, MAS, and EMA: Designed the study and made the final revision. All authors have read, reviewed, and approved the final manuscript.

## References

[1] Pierson T.C, Diamond M.S (2018). The emergence of Zika virus and its new clinical syndromes. Nature.

[2] Roehrig J.T (2013). West Nile virus in the United States-a historical perspective. Viruses.

[3] World Organization for Animal Health (OIE) Chapter 2.01.20:West Nile disease Manual of diagnostic tests and vaccines for terrestrial animals.

[4] Selim A, Abdelhady A (2020). The first detection of anti-West Nile virus antibody in domestic ruminants in Egypt. Trop. Anim. Health Prod..

[5] Domanović D, Gossner C.M, Lieshout-Krikke R, Mayr W, Baroti-Toth K, Dobrota A.M, Escoval M.A, Henseler O, Jungbauer C, Liumbruno G, Oyonarte S, Politis C, Sandid I, Vidović M.S, Young J.J, Ushiro-Lumb I, Nowotny N (2019). West Nile and Usutu virus infections and challenges to blood safety in the European union. Emerg. Infect. Dis..

[6] Richter J, Tryfonos C, Tourvas A, Floridou D, Paphitou N.I, Christodoulou C (2017). Complete genome sequence of West Nile virus (WNV) from the first human case of neuroinvasive WNV infection in Cyprus. Genome Announc..

[7] Cardinale E, Bernard C, Lecollinet S, Rakotoharinome V.M, Ravaomanana J, Roger M, Olive M.M, Meenowa D, Jaumally M.R, Melanie J, Héraud J.M, Zientara S, Cêtre-Sossah C (2017). West Nile virus infection in horses, Indian ocean. Comp. Immunol. Microbiol. Infect. Dis..

[8] Lwande O.W, Venter M, Lutomiah J, Michuki G, Rumberia C, Gakuya F, Obanda V, Tigoi C, Odhiambo C, Nindo F, Symekher S, Sang R (2014). Whole genome phylogenetic investigation of a West Nile virus strain isolated from a tick sampled from livestock in northeastern Kenya. Parasit. Vectors.

[9] Bondre V.P, Jadi R.S, Mishra A.C, Yergolkar P.N, Arankalle V.A (2007). West Nile virus isolates from India:Evidence for a distinct genetic lineage. J. Gen. Virol..

[10] Beck C, Lowenski S, Durand B, Bahuon C, Zientara S, Lecollinet S (2017). Improved reliability of serological tools for the diagnosis of West Nile fever in horses within Europe. PLoS Negl. Trop. Dis..

[11] Beck C, Jimenez-Clavero M.A, Leblond A, Durand B, Nowotny N, Leparc-Goffart I, Zientara S, Jourdain E, Lecollinet S (2013). Flaviviruses in Europe:Complex circulation patterns and their consequences for the diagnosis and control of West Nile disease. Int. J. Environ. Res. Public Health.

[12] Colpitts T.M, Conway M.J, Montgomery R.R, Fikrig E (2012). West Nile Virus:Biology, transmission, and human infection. Clin. Microbiol. Rev..

[13] Pierson T.C, Diamond M.S (2020). The continued threat of emerging flaviviruses. Nat. Microbiol..

[14] Carlson C.J, Albery G.F, Merow C, Trisos C.H, Zipfel C.M, Eskew E.A, Olival K.J, Ross N, Bansal S (2022). Climate change increases cross-species viral transmission risk. Nature.

[15] Bakonyi T, Ferenczi E, Erdélyi K, Kutasi O, Csörgo T, Seidel B, Weissenböck H, Brugger K, Bán E, Nowotny N (2013). Explosive spread of a neuroinvasive lineage 2 West Nile virus in Central Europe, 2008/2009. Vet. Microbiol..

[16] Shahhosseini N, Chinikar S, Moosa-Kazemi S.H, Sedaghat M.M, Kayedi M.H, Lühken R, Schmidt-Chanasit J (2017). West Nile Virus lineage-2 in Culex specimens from Iran. Trop. Med. Int. Health.

[17] Azmi K, Tirosh-Levy S, Manasrah M, Mizrahi R, Nasereddin A, Al-Jawabreh A, Ereqat S, Abdeen Z, Lustig Y, Gelman B, Schvartz G, Steinman A (2017). West Nile virus:Seroprevalence in animals in Palestine and Israel. Vector Borne Zoonotic Dis..

[18] Eybpoosh S, Fazlalipour M, Baniasadi V, Pouriayevali M.H, Sadeghi F, Vasmehjani A.A, Niya M.H.K, Hewson R, Salehi-Vaziri M (2019). Epidemiology of West Nile virus in the eastern Mediterranean region:A systematic review. PLoS Negl. Trop. Dis..

[19] Soliman A, Mohareb E, Salman D, Saad M, Salama S, Fayez C, Hanafi H, Medhat I, Labib E, Rakha M, El-Sayed N, Yingst S, Tjaden J, Earhart K (2010). Studies on West Nile virus infection in Egypt. J. Infect. Public Health.

[20] Selim A, Radwan A, Arnaout F, Khater H (2020). The recent update of the situation of West Nile fever among equids in Egypt after three decades of missing information. Pak. Vet. J..

[21] Calle-Tobón A, Holguin-Rocha A.F, Moore C, Rippee-Brooks M, Rozo-Lopez P, Harrod J, Fatehi S, Rua-Uribe G.L, Park Y, Londoño-Rentería B (2021). Blood meals with active and heat-inactivated serum modifies the gene expression and microbiome of *Aedes albopictus*. Front. Microbiol..

[22] Ziegler U, Angenvoort J, Klaus C, Nagel-Kohl U, Sauerwald C, Thalheim S, Horner S, Braun B, Kenklies S, Tyczka J, Keller M, Groschup M.H (2013). Use of competition ELISA for Monitoring of West Nile virus infections in horses in Germany. Int. J. Environ. Res. Public Health.

[23] Zeller H.G, Schuffenecker I (2004). West Nile virus:An overview of its spread in Europe and the Mediterranean basin in contrast to its spread in the Americas. Eur. J. Clin. Microbiol. Infect. Dis..

[24] Hou B, Chen H, Gao N, An J (2022). Cross-reactive immunity among five medically important mosquito-borne flaviviruses related to human diseases. Viruses.

[25] Papa A, Anastasiadou A, Delianidou M (2015). West Nile virus IgM and IgG antibodies three years post-infection. Hippokratia.

[26] Selim A, Megahed A, Kandeel S, Alouffi A, Almutairi M.M (2021). West Nile virus seroprevalence and associated risk factors among horses in Egypt. Sci. Rep..

[27] Bażanów B, van Vuren P.J, Szymański P, Stygar D, Frącka A, Twardoń J, Kozdrowski R, Pawęska J.T (2018). A survey on West Nile and Usutu viruses in horses and birds in Poland. Viruses.

[28] Durand B, Chevalier V, Pouillot R, Labie J, Marendat I, Murgue B, Zeller H, Zientara S (2002). West Nile virus outbreak in horses, Southern France, 2000:Results of a serosurvey. Emerg. Infect. Dis..

[29] Epp T, Waldner C, West K, Townsend H (2007). Factors associated with West Nile virus disease fatalities in horses. Can. Vet. J..

[30] Castillo-Olivares J, Wood J (2004). West Nile virus infection of horses. Vet. Res..

[31] Bunning M.L, Bowen R.A, Cropp C.B, Sullivan K.G, Davis B.S, Komar N, Godsey M.S, Baker D, Hettler D.L, Holmes D.A, Biggerstaff B.J, Mitchell C.J (2002). Experimental infection of horses with West Nile virus. Emerg. Infect. Dis..

[32] Sule W.F, Oluwayelu D.O, Hernández-Triana L.M, Fooks A.R, Venter M, Johnson N (2018). Epidemiology and ecology of West Nile virus in sub-Saharan Africa. Parasit. Vectors.

[33] Fang Y, Khater E.I.M, Xue J.B, Ghallab E.H.S, Li Y.Y, Jiang T.G, Li S.Z (2022). Epidemiology of mosquito-borne viruses in Egypt:A systematic review. Viruses.

[34] Mohamed R.A.E, Abdelgadir D.M, Bashab H.M, Al-Shuraym L.A, Aleanizy F.S, Alqahtani F.Y, Al-Keridis L.A, Mohamed N (2020). First record of West Nile virus detection inside wild mosquitoes in Khartoum capital of Sudan using PCR. Saudi J. Biol. Sci..

